# The safe use of inflammatory bowel disease therapies during the COVID-19 pandemic

**DOI:** 10.1016/j.crphar.2022.100101

**Published:** 2022-04-26

**Authors:** Chethana Kamath, Erica J Brenner

**Affiliations:** Division of Pediatric Gastroenterology, Department of Pediatrics, University of North Carolina at Chapel Hill, Chapel Hill, NC, USA

**Keywords:** 5-ASA, 5-aminosalicylates, AGA, American Gastrointestinal Association, aOR, adjusted odds ratio, CI, confidence interval, COVID-19, coronavirus disease 2019, ICU, IL, interleukin, intensive care unit, IBD, Inflammatory Bowel Disease, JAK, janus kinase inhibitor, MTX, methotrexate, SECURE-IBD, Surveillance Epidemiology of Coronavirus Under Research Exclusion for Inflammatory Bowel Disease, TNF, tumor necrosis factor, UC, Ulcerative Colitis

## Abstract

**Background:**

Patients with inflammatory bowel disease (IBD) often require the use of immunosuppressant medications that increase infection risk, leading to concerns over the safe use of IBD medications during the Coronavirus 19 (COVID-19) pandemic.

**Objectives:**

To summarize available evidence on the safety and appropriate use of IBD medications during the COVID-19 pandemic, particularly in regard to risk of severe COVID-19 outcomes such as hospitalization, respiratory failure, or death for patients on IBD therapeutics.

**Conclusions:**

The majority of IBD medications are safe to continue during the COVID-19 pandemic, with a few notable exceptions. Patients with IBD who do not have COVID-19 should continue their prescribed IBD therapies, although steroids are associated with severe COVID-19 outcomes and should be weaned when possible. Corticosteroids should be tapered and discontinued when possible in patients with IBD who test positive for COVID-19 as well. Patients with IBD who test positive for COVID-19 should hold biologics, thiopurines, methotrexate, and tofacitinib for at least 2 weeks, and those who have symptoms should not restart these medications until symptom resolution. During the COVID-19 pandemic, all patients with IBD should continue to follow public health guidance including social distancing, masking, and COVID-19 vaccination recommendations.

## Highlights

• Asymptomatic IBD patients with COVID-19 should hold biologics and certain immunomodulators for at least 2 weeks.
• Symptomatic IBD patients with COVID-19 should hold biologics and certain immunomodulators until symptoms are improved.
• Corticosteroids should be tapered and discontinued when possible in patients with IBD who test positive for COVID-19.

## 1 Introduction

Inflammatory bowel disease (IBD) is a chronic inflammatory condition that affects over a million people worldwide (Ng et al., 2018; Ungaro et al., 2017; Torres et al., 2017). The treatment for IBD often necessitates immunosuppressant medications that can simultaneously increase the risk for bacterial and viral infections (Rahier et al., 2014; Kirchgesner et al., 2018; Long et al., 2013; Tinsley et al., 2019). In December 2019, it was first reported that a virus called SARS-CoV-2 was causing COVID-19, a disease process causing a range of presentations from asymptomatic infection to severe cases leading to hospitalization, respiratory failure, or death (Onder et al., 2020; Wu and McGoogan, 2020). The Center for Disease Control also defined risk factors for severe infection as use of steroids or other immune system weakening medications as well as presence of an underlying medical condition (Center for Disease Control, 2021). This led to concerns during the COVID-19 pandemic that patients with IBD would be at risk for severe outcomes from the virus due to immunosuppressive agent use.

In response to this concern early in the pandemic, the American Gastroenterological Association (AGA) created a clinical practice update to aid clinicians in management of IBD during the COVID-19 Pandemic that included expert commentary and guidance (Rubin et al., 2020). As the pandemic has continued, gastroenterology providers and researchers worldwide have collaborated to gather data on the impact of COVID-19 on patients with IBD, including the association between IBD treatments and COVID-19 outcomes. The Surveillance Epidemiology of Coronavirus Under Research Exclusion for Inflammatory Bowel Disease (SECURE-IBD) database, a large, international registry of IBD patients with COVID-19, provides an important example of such international cooperation (Brenner et al., 2020). In this review, we summarize recommendations for the safe use of IBD medications during the COVID-19 pandemic, based on literature from the SECURE-IBD registry and other relevant studies. While this review provides general guidance for IBD medical management during the pandemic, the care of patients with IBD should be individualized and made in collaboration with the patient's IBD provider.

## 2 Associations between IBD medications and COVID-19 outcomes

### 2.1 Mesalamine/sulfasalazine

There is no statistically significant association between mesalamine/sulfasalazine use and any adverse COVID-19 outcome among patients with IBD (Ungaro et al., 2022, Ungaro et al., 2021b). Initial SECURE-IBD analyses found an increased risk of COVID-19 outcomes associated with this subgroup when compared to other medications (Brenner et al., 2020). However, this association has attenuated over time, likely related to reporting bias and/or reporting delays (Ungaro et al., 2021). Lack of a dose-response relationship further supports the conclusion that sulfasalazine/mesalamine are not associated with increased risk of poor COVID-19 outcomes (Ungaro et al., 2022, Ungaro et al., 2021b). A population-based study of patients with ulcerative colitis (UC) in Denmark similarly found that patients treated with mesalamine/sulfasalazine had no increased risk for COVID-19-related hospitalization (Kjeldsen et al., 2021). Mesalamine/sulfasalazine is safe to continue in asymptomatic and symptomatic patients with COVID-19 (Rubin et al., 2020).

### 2.2 Systemic steroids

Systemic steroids were significantly associated with increased odds of hospitalization, severe COVID-19 (defined as intensive care unit [ICU] stay, hospitalization and/or death), and death (Brenner et al., 2020; Ungaro et al., 2022, Ungaro et al., 2021b). Most recent literature suggests adjusted odds of COVID-19-related hospitalization as 2.45 (95% CI 1.81–3.31), with severe COVID-19 aOR 3.49 (95% CI 2.62–4.65) and death aOR 4.77 (95% CI 3.36–6.77) (Ungaro et al., 2022, Ungaro et al., 2021b). These effects are thought to occur when steroids are given prior to the time of infection before the onset of a cytokine storm leading to deleterious effects on viral clearance or ability to mount a proper immune response (Russell et al., 2020). Therefore, these findings do not contradict current trials demonstrating a mortality benefit of short-term corticosteroids (Mahase, 2020), likely by blunting a hyperimmune response in severely ill patients (Russell et al., 2020).

### 2.3 Budesonide

There are limited studies evaluating the safety of budesonide use in patients with IBD during the COVID-19 pandemic, and analyses from the SECURE-IBD registry remain underpowered to evaluate COVID-19 outcomes associated with budesonide use (Ungaro et al., 2022, Ungaro et al., 2021b). One retrospective multi-center cohort study compared budesonide to prednisolone in UC patients in 2019 and 2020. It was discovered that after week four of treatment there was average increase in bowel frequency (3.49 in 2019 vs 5.85 in 2020, p ​= ​0.001) and rectal bleeding (in 89.7% of patient in 2019 and 73.1% in 2020, p ​= ​0.039) but there was no significant difference in rectal bleeding and hospital admissions at week eight for patients on budesonide compared to prednisolone (Rosiou et al., 2021).

### 2.4 Biologics and immunomodulators

Multiple cohort studies have found no increased risk of severe COVID-19 associated with any of the biologic classes, including Tumor Necrosis Factor (TNF) antagonist agents, integrin antagonists, and interleukin (IL)-12/23 inhibitors (Ungaro et al., 2022, Ungaro et al., 2021b; Lukin et al., 2020; Agrawal et al., 2021). In studies from the SECURE-IBD registry, TNF antagonist agents had a decreased odds of COVID-19 related hospitalization, severe COVID-19, and death (Ungaro et al., 2022, Ungaro et al., 2021b). These findings may suggest a protective effect (Ungaro et al., 2022, Ungaro et al., 2021b) due to blunting of the robust inflammatory response seen in severe disease, but randomized trial data is needed for further evaluation (Feldmann et al., 2020; Rizk et al., 2020). A few of these medications (adalimumab and infliximab) are being studied currently in randomized control trials as a potential treatment for COVID-19 (Rizk et al., 2020). Integrin antagonists were associated with decreased odds of COVID-19-related hospitalizations (aOR 0.66, 95% CI 0.56–0.78) (Ungaro et al., 2022, Ungaro et al., 2021b). IL-12/23 Inhibitors had decreased odds of COVID-19-related hospitalization (aOR 0.44, 95% CI 0.36–0.54) and severe COVID-19 (aOR 0.43, 95% CI 0.26–0.71) (Ungaro et al., 2022, Ungaro et al., 2021b). Both integrin antagonists and IL-12/23 inhibitors have a risk profile similar to TNF antagonist monotherapy (Ungaro et al., 2022, Ungaro et al., 2021b). In psoriasis, another autoimmune condition where IL-12/23 inhibitors are commonly used, these medications were associated with a decreased risk of COVID-19 related hospitalization (Kridin et al., 2021).

Thiopurine monotherapy is associated with a significantly elevated risk of severe COVID-19 (aOR 4.08, 95% CI 1.73 to 9.61) (Ungaro et al., 2022, Ungaro et al., 2021b). TNF antagonist and thiopurine combination therapy is associated with a significantly increased odds of hospitalization and/or death (aOR 1.82, 95% CI 1.26–2.62) but not severe COVID-19 (aOR 1.63, 95% CI 0.87–3.10) (Ungaro et al., 2022, Ungaro et al., 2021b). This finding aligns with previous literature showing a higher risk of viral infections in individuals who take thiopurines alone or in combination (Kirchgesner et al., 2018). In the most recent analyses, methotrexate (MTX) monotherapy had no association with death (aOR 0.79 (0.2–3.08) or severe COVID, just a trend toward increased hospitalization (Ungaro et al., 2022, Ungaro et al., 2021b). TNF antagonist and MTX use were not associated with increased odds of hospitalization and/or death (aOR 0.82, 95% CI 0.42–1.60 or severe COVID-19 (OR 2.44, 95% CI 0.55–10.74) (Ungaro et al., 2022, Ungaro et al., 2021b), which is consistent with previous literature regarding infection risk associated with these medications Agrawal et al. (2021); Baradat et al. (2017). In the SECURE-IBD database, tofacitinib (a Janus kinase [JAK] inhibitor) was not associated with an elevated risk of severe COVID-19, nor was it associated with an increased risk of thrombotic events in patients with IBD who developed COVID-19 (Agrawal et al., 2021). Several JAK inhibitors are currently being studied (including tofacitinib) as potential COVID-19 treatments (Rizk et al., 2020).

## 3 IBD medication management recommendations during the COVID-19 pandemic

For patients with IBD that have not been infected with COVID-19, avoiding disease exacerbation should remain a primary goal, and therefore most patients should remain on their current medication regimen, with exceptions as noted in the Table 1 (Rubin et al., 2020). All patients with IBD should follow public health guidance during the pandemic, including adhering to strict social distancing recommendations, masking, practicing good hygiene, and receiving a COVID-19 vaccination. Infusion of needed therapies should continue, and it is not recommended to switch to injectable therapies as this has been found to be associated with relapses (Van Assche et al., 2012). Home infusions are also not recommended as this can lead to swifter transmission from home nurses as vectors to patients (Rubin et al., 2020).

For patients with IBD that are asymptomatic but sero-positive for COVID-19, oral steroids should be reduced to lower than <20 ​mg/day of prednisone or transitioned for budesonide if possible (Rubin et al., 2020). Therapies such as 5-ASA, budesonide, rectal therapies, and enteral nutrition should be continued. Other medications such as methotrexate, thiopurine, and tofacitinib should be held temporarily. Biologics such as anti-TNF therapies, ustekinumab, and vedolizumab should be delayed for two weeks to monitor for development of symptoms of COVID-19. If the patient does not develop any symptoms, it is reasonable to re-start monoclonal therapy after that period or if repeat viral testing is negative (Rubin et al., 2020). Patients with mild COVID-19 symptoms not requiring hospitalization should adhere to the above recommendations as well (Rubin et al., 2020).

For patients with IBD that develop moderate COVID-19 symptoms, including those requiring hospitalization, particularly with hypoxia or imaging evidence of pneumonia or severe disease (required respiratory support, needing pressors, or organ damage), treatment should focus on addressing the underlying acute COVID-19 illness with anti-inflammatory, anti-cytokine and/or anti-viral therapies as indicated while following guidelines for management of hospitalized IBD patients (Kaur et al., 2020). If intravenous corticosteroids are needed, the course should be limited to three days and switching to a calcineurin inhibitor or infliximab should be considered (Rubin et al., 2020). Systemic corticosteroids should otherwise be avoided and tapered off with special attention to the possibility of adrenal insufficiency from chronic use (Rubin et al., 2020; Targownik et al., 2021). Mesalamine/sulfasalazine, budesonide, rectal therapies, and enteral nutrition are considered appropriate to continue in the setting of moderate COVID-19 symptoms. Thiopurines, methotrexate and tofacitinib should be stopped. Anti-TNF therapies, ustekinumab, and vedolizumab should be delayed for at least 2 weeks to monitor for symptom resolution but can be re-started once symptoms resolve, convalescent COVID-19 titers develop, or sequential viral testing is negative (Rubin et al., 2020). See Table 1 for a detailed list of IBD medication safety and management considerations during the COVID-19 pandemic.

**Table 1:** Safety and management recommendations of IBD therapy during COVID-19 pandemic.

	General Safety Recommendation During COVID-19 Pandemic	Management Recommendations During COVID-19 Pandemic
Sulfasalazine/Mesalamine	This medication is SAFE to continue during the COVID-19 pandemic.	This medication is safe to continue in asymptomatic and symptomatic patients with COVID-19 (Rubin et al., 2020).
Oral/Parenteral Steroids	This medication increases risk of adverse COVID-19 outcomes.	Corticosteroids should be used judiciously and weaned as soon as possible. IBD patients affected by COVID-19 should have their corticosteroids held or tapered to the minimal effective dose when possible (El Ouali et al., 2021; Rubin et al., 2020). For patients with IBD who test positive for COVID-19 but who are asymptomatic, their prednisone dose should be reduced to less than <20 ​mg/day or changed to budesonide if possible (Rubin et al., 2020). In cases with patients who are hospitalized with mild COVID-19, it is suggested that IV steroids should be limited to 3 days and proceeding with a calcineurin inhibitor or infliximab should be considered (Rubin et al., 2020).
Budesonide	This medication is SAFE to continue during the COVID-19 pandemic in patients with IBD who do not test positive for COVID-19.	Budesonide is safe to continue during the pandemic if it is already being used to maintain remission of IBD. In circumstances where a patient with IBD is on a systemic corticosteroid and asymptomatic or has mild COVID-19 symptoms, switching to budesonide should be considered as an option (Rubin et al., 2020; Rosiou et al., 2021).
Thiopurine Monotherapy	This medication is SAFE to continue during the COVID-19 pandemic in patients with IBD who do not test positive for COVID-19.	Patients with IBD who test positive for COVID-19 should hold this medication until their symptoms are resolved (if symptomatic) or repeat viral testing is negative (Rubin et al., 2020).
Methotrexate Monotherapy	This medication is SAFE to continue during the COVID-19 pandemic in patients with IBD who do not test positive for COVID-19.	Patients with IBD who test positive for COVID-19 should hold this medication until their symptoms are resolved (if symptomatic) or repeat viral testing is negative (Rubin et al., 2020).
TNF Antagonist Monotherapy	This medication is SAFE to continue during the COVID-19 pandemic in patients with IBD who do not test positive for COVID-19.	Patients with IBD who test positive for COVID-19 hold this medication until their symptoms are resolved (if symptomatic) or repeat viral testing is negative. Re-starting biologics should be delayed for at least 2 weeks to monitor development of COVID-19 symptoms (Rubin et al., 2020).
TNF Antagonist with Thiopurines	In select high-risk patients (i.e older age or multiple comorbidities) in stable remission with TNF antagonist combination therapy, consider discontinuing the thiopurine during the COVID-19 pandemic, particularly if a patient is at high risk for COVID-19 exposure and/or unvaccinated (Ungaro et al., 2022, Ungaro et al., 2021b).	Patients with IBD who test positive for COVID-19 should hold these medications until their symptoms are resolved (if symptomatic) or repeat viral testing is negative. Re-starting biologics should be delayed for at least 2 weeks to monitor development of COVID-19 symptoms (Rubin et al., 2020).
TNF Antagonist with MTX	This combination of medications is SAFE to continue during the COVID-19 pandemic in patients with IBD who do not test positive for COVID-19.	Patients with IBD who test positive for COVID-19 should hold these medications until their symptoms are resolved or repeat viral testing is negative. Re-starting biologics should be delayed for at least 2 weeks to monitor development of COVID-19 symptoms (Rubin et al., 2020).
Integrin Antagonists	This medication is SAFE to continue during the COVID-19 pandemic in patients with IBD who do not test positive for COVID-19.	Patients with IBD who test positive for COVID-19 should hold this medication until their symptoms are resolved or repeat viral testing is negative. Re-starting biologics should be delayed for at least 2 weeks to monitor development of COVID-19 symptoms (Rubin et al., 2020).
Interleukin (IL)-12/23 Inhibitor	This medication is SAFE to continue during the COVID-19 pandemic in patients with IBD who do not test positive for COVID-19.	Patients with IBD who test positive for COVID-19 should hold this medication until their symptoms are resolved (if symptomatic) or repeat viral testing is negative. Re-starting biologics should be delayed for at least 2 weeks to monitor development of COVID-19 symptoms (Rubin et al., 2020).
JAK Inhibitor	This medication is SAFE to continue during the COVID-19 pandemic if the patient has not tested positive for COVID-19.	Patients with IBD who test positive for COVID-19 should hold this medication until their symptoms are resolved (if symptomatic) or repeat viral testing is negative (Rubin et al., 2020).

## 4 Conclusion

Patients with IBD, a chronic and life-threatening condition, can have a spectrum of disease presentations during the COVID-19 pandemic whether asymptomatic or leading to hospitalization, respiratory failure, or death (Onder et al., 2020; Wu and McGoogan, 2020). It is crucial to prioritize proper control of IBD for these patients. As a result, most patients should continue their present pharmaceutical regimen during the pandemic. Notable exceptions include weaning corticosteroids when possible and briefly holding biologics and immunomodulators if a patient becomes infected by COVID-19 (Rubin et al., 2020). During the COVID-19 pandemic, all patients with IBD should continue to follow public health recommendations, which include social distancing, proper sanitation practices and obtaining vaccinations (Center for Disease Control, 2021).

## CRediT authorship contribution statement

**Chethana Kamath:** Conceptualization, Visualization, Methodology, Investigation, Data curation, Writing – original draft, Writing – review & editing. **Erica J Brenner:** Conceptualization, Project administration, Resources, Supervision, Writing – review & editing.

## Declaration of competing interest

The authors declare the following financial interests/personal relationships which may be considered as potential competing interests:

Dr. Kamath and Dr. Brenner report no conflict of interest. Dr Brenner is supported by a T32 training grant from the 10.13039/100000002National Institutes of Health (grant number T32 DK007634).
